# CKD-506, a novel HDAC6-selective inhibitor, improves renal outcomes and survival in a mouse model of systemic lupus erythematosus

**DOI:** 10.1038/s41598-018-35602-1

**Published:** 2018-11-23

**Authors:** Eun Wha Choi, Ji Woo Song, Nina Ha, Young Il Choi, Sungjoo Kim

**Affiliations:** 1 Department of Veterinary Clinical Pathology, College of Veterinary Medicine & Institute of Veterinary Science, Kangwon National University, 1 Kangwondaehak-gil, Chuncheon, Gangwon-do 24341 Republic of Korea; 2 Transplantation Research Center, Samsung Biomedical Research Institute, Samsung Medical Center, 81 Irwon-ro, Gangnam-gu, Seoul, 06351 Republic of Korea; 3 CKD Research Institute, 315-20 Dongbaek Jukjeon-Daero, Yongin, 16995 Republic of Korea; 4 Department of Surgery, Division of Transplantation, Samsung Medical Center, Sungkyunkwan University School of Medicine, 81 Irwon-ro, Gangnam-gu, Seoul, 06351 Republic of Korea

## Abstract

Systemic lupus erythematosus (SLE) is a chronic multisystemic autoimmune disease with an unknown etiology. Recently, it has been elucidated that dysregulated histone deacetylase (HDAC) activity is related to the pathogenesis of inflammatory and autoimmune diseases. Broad-spectrum HDAC inhibitors are effective for the treatment of allergy, cancer, and autoimmune diseases, but they have several adverse side effects. Thus, the purpose of this study was to evaluate the effects of a novel HDAC 6-specific inhibitor, CKD-506, in a murine SLE model. CKD-506 significantly improved survival rate and significantly decreased the incidence of severe proteinuria, blood urea nitrogen, kidney inflammation, and glomerular infiltration of IgG and C3. In addition, CKD 506 reduced the proportions of CD138^+^ plasma cells, CD4^−^CD8^−^ T cells, and CD25^+^ cells and the Th1/Th2 ratio in the spleen. CKD-506 significantly reduced inflammatory cytokines such as IL-10, IL-15, IL-17, TNF-α, and IFN-inducible protein (IP-10) and significantly increased TGF-β in serum. CKD-506 also significantly reduced IFN-γ, IL-1β, IL-4, IL-6, IP-10, MCP-1, and CCL4 levels in kidney. CKD-506 decreased the production of various pro-inflammatory cytokines and chemokines in the serum and kidneys, resulting in inhibition of cell migration and suppression of lupus nephritis without adverse effects.

## Introduction

Systemic lupus erythematosus (SLE) is a chronic multisystemic autoimmune disease that occurs when body tissues and organs are attacked by its own immune system; in SLE, anti-nuclear antibodies are developed, and then circulating antigen-antibody complexes are produced and lodge in small vessels and various organ systems, especially in the basement membrane zone of the skin and kidney. Circulating antigen-antibody complexes activate the complement cascade and facilitate inflammatory cell accumulation, resulting in various tissue inflammation^1^. Deposition of immune complexes within the glomerulus leads to glomerulonephritis, which called lupus nephritis. Kidney failure is one of the leading causes of death among patients with lupus^2^. One of the most commonly used animal models for SLE is the (NZB × NZW) F1 (NZB/W F1) female mouse. NZB/W F1 female mice show a spontaneous autoimmune disease process similar to the pathogenesis of human SLE; they produce anti-nuclear antibodies, including anti-double-stranded (ds) DNA antibodies, and develop severe immune complex-mediated glomerulonephritis. These mice die of renal failure by the age of 10–12 months^3^.

Corticosteroid, antimalarials, and immunosuppressive drugs are the basis for SLE therapy and are currently used^4^. These drugs may be effective in many cases of SLE patients, but they are associated with substantial toxicities and are not uniformly efficacious. Treatment of patients with active SLE refractory to traditional therapies continues to be difficult^4^.

The exact etiology of SLE is unknown, but complex interactions among genetic factors, inappropriate immune regulation, and other factors, such as hormonal and environmental variables, are thought to cause SLE. Epigenetic regulatory defects such as abnormal DNA methylation, miRNA regulation, and histone modifications have also recently been suggested to contribute to SLE^5^. Dysregulated histone deacetylase (HDAC) activity is related to the pathogenesis of inflammatory and autoimmune diseases^6–10^.

Histone acetylation plays an important role in gene expression; acetylation generally results in increased transcription, while deacetylation is associated with gene repression^11^. Pan HDAC inhibitors showed excellent efficacy in the treatment of allergy, cancer, and autoimmune diseases^12–14^. However, their significant adverse effects such as fatigue, anorexia, nausea, vomiting, diarrhea, weight loss, asthenia, thrombocytopenia, neutropenia, anemia, and alteration of serum biochemistry profiles significantly limited its clinical application in chronic indications such as SLE^12^. Thus, it has been proposed that HDAC subtype selective inhibitors, which have fewer adverse effects than Pan HDAC inhibitors, can be used for treatment of chronic diseases; the emerging trend is to identify HDAC isozyme selective inhibitor with both immunomodulatory activity and improved safety profile.

HDAC 6, a cytoplasmic class IIb HDAC, deacetylates nonhistone proteins including heat shock protein (HSP 90) and α-tubulin, and regulates protein degradation^11,13^. HDAC6 plays a critical role in the formation of immune synapses and modulation of immune responses^15^. A recent *in vitro* study revealed that overexpression of HDAC6 significantly increased expression of pro-inflammatory cytokines such as TNF-α, IL-1β, and IL-6 by upregulating NF-kB and AP-1 signaling pathways in macrophages^16^. Thus, we hypothesized that a novel HDAC 6 inhibitor, CKD-506, might improve the symptoms of SLE by reducing the production of various lupus disease-specific cytokines and chemokines.

In this study, we evaluated the therapeutic efficacy of a novel HDAC 6 inhibitor, CKD-506, in a murine SLE model, NZB/W F1 female mice.

## Results

### CKD-506 is a potent and selective HDAC6 inhibitor

**Figure 1:**
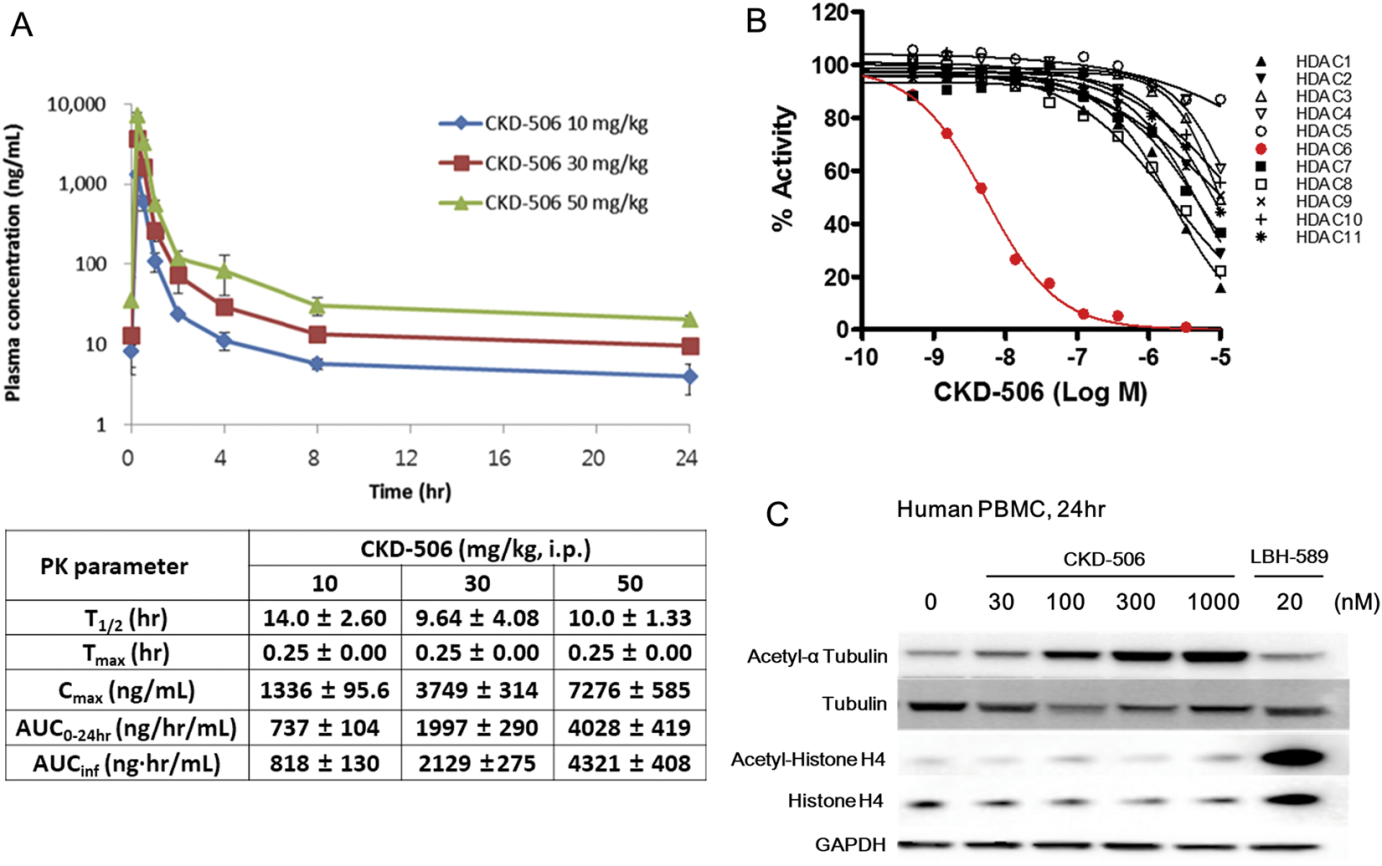
Enzyme selectivity of CKD-506 & Pharmacokinetic profile of CKD-506. The selectivity of CKD-506 was analyzed based on HDAC enzyme inhibition and protein acetylation. (**A**) After single intraperitoneal injection of CKD-506, blood samples were collected at the indicated time points. The plasma concentration of CKD-506 was analyzed. All the data were presented as mean ± SD. Upper panel: plasma concentration – time plot of CKD-506; Lower table: pharmacokinetic parameters of CKD-506. (**B**) The enzyme activity of HDAC family members with the indicated concentration of CKD-506 (log of molar concentration; Log M) was analyzed and plotted. The Y-axis represents the percentage of enzymatic activity normalized to the enzyme activity without CKD-506. (**C**) The HDAC6 enzyme selectivity of CKD-506 in human PBMC was analyzed based on the acetylation of α tubulin, one of the major target proteins of HDAC6, and the acetylation of histone H4 which is not a target protein of other HDAC isozymes. LBH-589 is a pan HDAC inhibitor, a positive control for the induction of histone H4 acetylation (the chemicals were treated for 4 hr.). Samples were derived from the same experiment, and blots were processed in parallel. Full-length blots are presented in Supplementary Fig. 1. T_1/2_: elimination half-life; hr: hour; T_max_: time to maximum plasma concentration; Cmax: maximum plasma concentration; AUC_0–24hr_: area under the curve from time zero to 24 hr; AUC_inf_: area under the curve from time zero to infinity (extrapolated data); i.p.: intraperitoneal.

In HDAC panel assay, CKD-506 inhibited HDAC6 selectively with IC_50_ value of around 5 nM. IC_50_ values for HDAC1, HDAC2, HDAC7 and HDAC8 were in the range of 2000–5000 nM. CKD-506 does not inhibit enzyme activity of other HDAC isoforms (Fig. 1A). To confirm the intracellular inhibitory activity of CKD-506, the effect of CKD-506 on the acetylation of tubulin, a major HDAC6 target protein, and histone H4 was analyzed with human PBMC (Fig. 1B). CKD-506 induced the acetylation of α tubulin from 30 nM without affecting the acetylation of histone H4 which is not a target protein of HDAC6. This result confirmed that CKD-506 is a potent and selective HDAC6 inhibitor.

### Pharmacokinetic profile of CKD-506

To evaluate the relationship between the dose and efficacy of CKD-506 on SLE with NZB/W F1 mice, pharmacokinetic profile was analyzed with the same NZB/W F1 mice after single intraperitoneal injection (IP) of CKD-506 (Fig. 1A). The plasma half-life (T_1/2_) of CKD-506 was about 10 hrs and the plasma concentration of CKD-506 reached the maximum (Cmax) in about 30 min after IP of CKD-506. The expose of CKD-506 (maximum plasma concentration (Cmax) and area under the curve (AUC)) was increased in a dose-dependent manner. Area under the curve from time zero to 24 hr (AUC_0–24hr_) of CKD-506 at each dose was comparable to AUC from time zero to infinity (AUC_inf_). In 50 mg/kg group, the plasma concentration of CKD-506 was around 200 nM (84.5 ng/ml) even in 4 hr of IP injection of CKD-506. When considering the fact that CKD-506 inhibited enzyme activity over 90% from 100 nM (Fig. 1B) and induced the acetylation of α tubulin at 100 nM significantly (about 85% of maximum acetylation of α tubulin) (Fig. 1C), 50 mg/kg of CKD-506 is enough to inhibit 90% of HDAC6 activity for more than 4 hr.

### Survival rates

**Figure 2:**
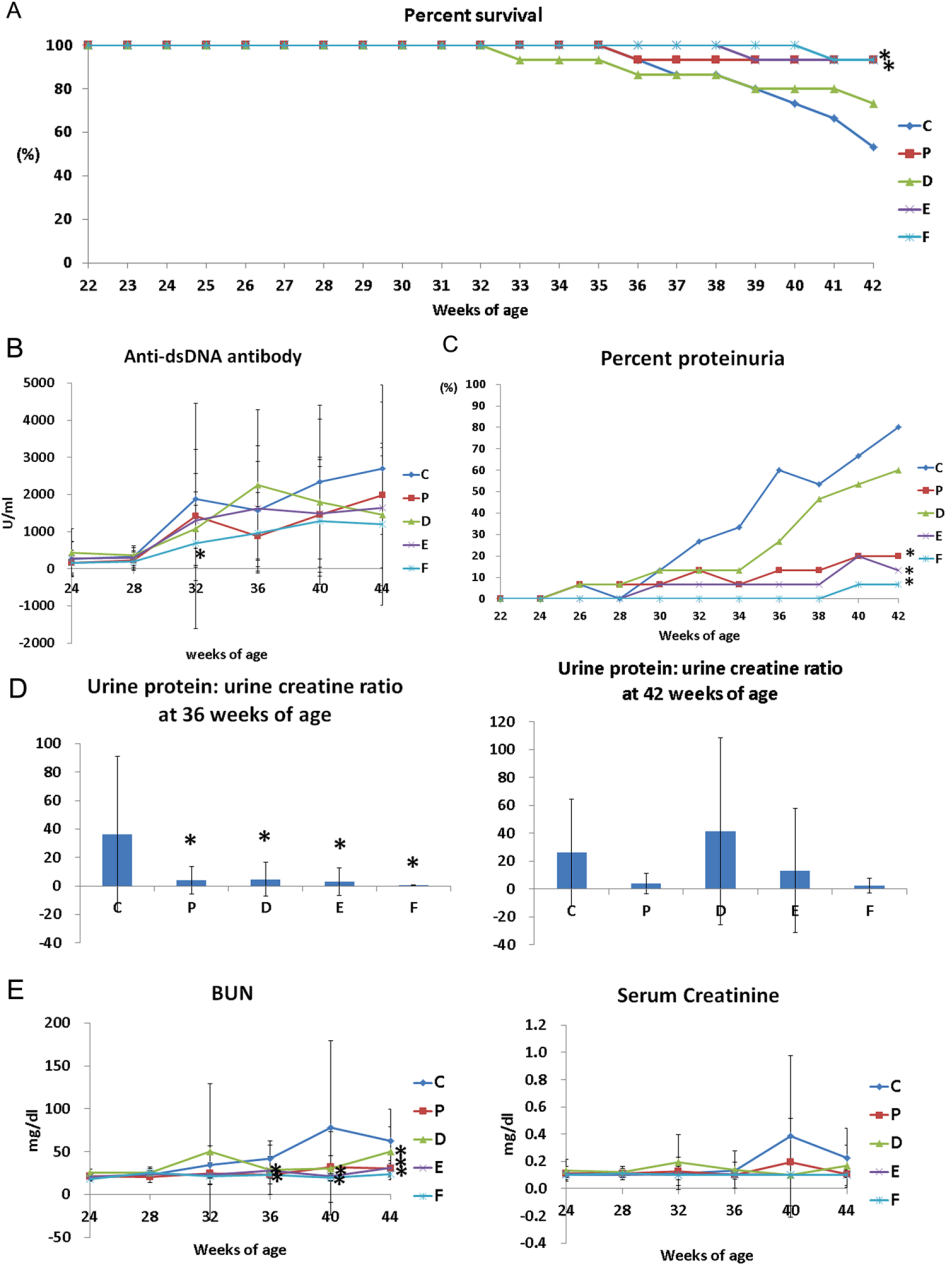
Survival, progression of severe proteinuria, and serology. (**A**) Survival, (**B**) serum levels of anti-dsDNA antibodies, (**C**) incidence of severe proteinuria (≥300 mg/dl), (**D**) urine protein/urine creatinine ratio, and (**E**) concentrations of BUN and creatinine in (NZB × NZW) F1 mice after CKD-506 treatment. Data from A and C were analyzed using Kaplan-Meier curves and a log rank test. The significance of differences in serums levels of anti-dsDNA antibodies between pairwise combinations of the treatment groups and control group was assessed by t-test. For other data, intergroup analysis was performed using ANOVA followed by Tukey’s multiple comparison *post-hoc* tests. *Significant difference (*p* < 0.05) from the control (C group). C: control group (vehicle (Ethanol: Kolliphor® EL: saline = 1:1:8) 150 μl), P: methylprednisolone (5 mg/kg/day) treatment group, D: CKD-506, 10 mg/kg/day, E: CKD-506, 30 mg/kg /day, F: 50 mg/kg/day treatment group.

Survival rates of groups vehicle, methylprednisolone, CKD10, CKD30, and CKD50 at the end of the study (42-weeks of age) were 53.3%, 93.3%, 73.3%, 93.3%, and 93.3%, respectively. Survival rate was significantly higher in groups CKD30 and CKD50 than in group vehicle (log-rank test, *p* = 0.026, C vs. E: *p* = 0.016, C vs. F: *p* = 0.014, Fig. 2A).

### Anti-dsDNA antibody concentrations

Groups methylprednisolone, CKD30, and CKD50 had significantly lower mean anti-dsDNA antibody values than group vehicle, but this was not statistically significant (ANOVA: p > 0.05). In pairwise comparisons among groups, anti-dsDNA antibody concentrations at 32 weeks were significantly lower in group CKD50 than in group vehicle (t-test: *p* = 0.011, Fig. 2B).

### Incidence of severe proteinuria

The incidence of severe proteinuria (urine protein >300 mg/dl) at 42 weeks of age was 80% in group vehicle, 20% in group methylprednisolone, 13.3% in group CKD10, 6.7% in group CKD30, and 0% in group CKD50. Incidence of severe proteinuria was significantly lower in groups methylprednisolone, CKD30, and CKD50 than in group vehicle (log-rank test, *p* > 0.001, vehicle vs. methylprednisolone: *p* = 0.002, vehicle vs. CKD10: *p* = 0.191, vehicle vs. CKD30: *p* = 0.001, vehicle vs. CKD50: *p* < 0.001, Fig. 2C).

When the urinary protein to creatinine ratio was measured at 36 weeks, urine protein/urine creatinine (UP/C) was significantly lower in the other treatment groups than in group vehicle (ANOVA & Tukey, P = 0.006). At 42 weeks, group CKD50 had the lowest UP/C mean value (Fig. 2D), but this was not significantly different than the UP/C values in the other groups.

### BUN and serum creatinine concentrations

Levels of BUN at 36 weeks of age in groups methylprednisolone and CKD50 were significantly lower than those in group C (ANOVA & Tukey, *p* = 0.038). Levels of BUN at 40 weeks of age in groups CKD30 and CKD50 were significantly lower than those in group vehicle (*p* = 0.018). Levels of BUN at autopsy were significantly lower in groups methylprednisolone, CKD30, and CKD50 than in group vehicle (*p* < 0.001, Fig. 2E).

### Histopathological lesions of the kidneys

**Figure 3:**
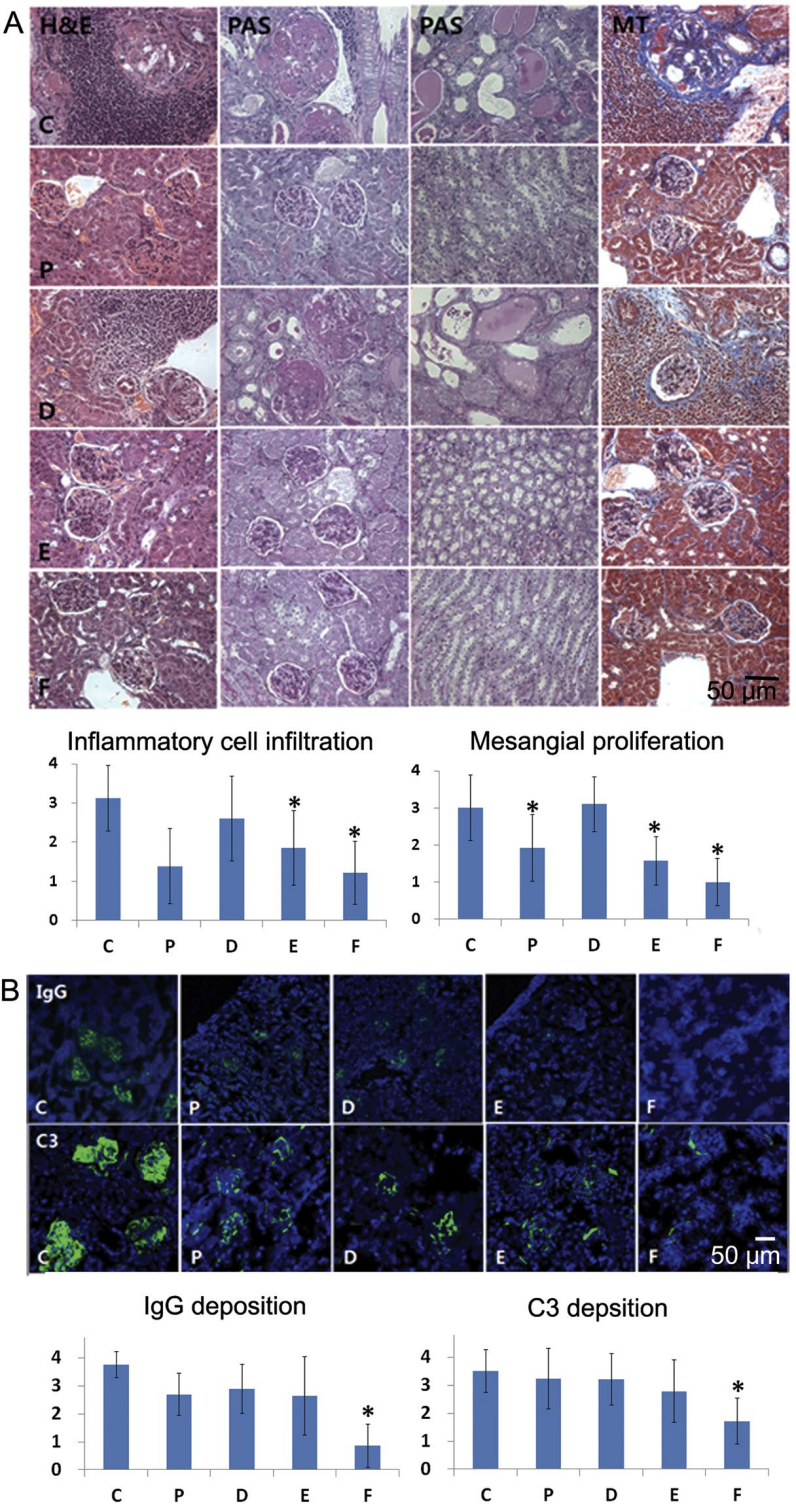
Histopathology and immunofluorescence analysis of kidneys. (**A**) Hematoxylin and eosin (H&E), periodic acid-Schiff (PAS) reagent, and Masson’s trichrome staining of kidneys obtained from mice at the end of the experiment (original magnification: ×400). Inflammatory cell infiltration and mesangial proliferation were scored on a graded scale from 0 (no) to 4 (severe). (**B**) IgG and C3 deposition in kidneys (original magnification: ×200). Fluorescence staining intensities of IgG and C3 deposits were graded as 0 (none), 1+ (mild), 2+ (moderate), 3+ (moderate to strong), or 4+ (strong). Intergroup analysis was performed using ANOVA followed by Tukey’s multiple comparison *post-hoc* tests. *p < 0.05 (vs. C). C: control group (vehicle (ethanol: Kolliphor® EL: saline = 1:1:8) 150 μl), P: methylprednisolone (5 mg/kg/day) treatment group, D: CKD-506, 10 mg/kg/day, E: CKD-506, 30 mg/kg/day, F: 50 mg/kg/day treatment group.

Large numbers of inflammatory cell infiltrations and mesangial proliferation were observed in groups vehicle and CKD10 on histopathological evaluation of the kidneys (Fig. 3A). Degree of inflammatory cell infiltration was significantly lower in groups methylprednisolone, CKD30, and CKD50 than in group vehicle (ANOVA & Tukey, *p* < 0.001). Periodic acid-Schiff (PAS) staining revealed dilated tubules and casts in groups vehicle and CKD10, and Masson’s trichome straining revealed fibrosis in groups vehicle and CKD10 (Fig. 3A).

### IgG and C3 infiltration in the kidneys

When the degrees of IgG and C3 infiltration in glomeruli were evaluated, they were found to be significantly lower in group CKD50 than group vehicle (ANOVA & Tukey, IgG: *p* < 0.001 and C3: *p* < 0.001, Fig. 3B).

### Flow cytometric determination of the T cell and T helper subset proportions in the spleen

**Figure 4:**
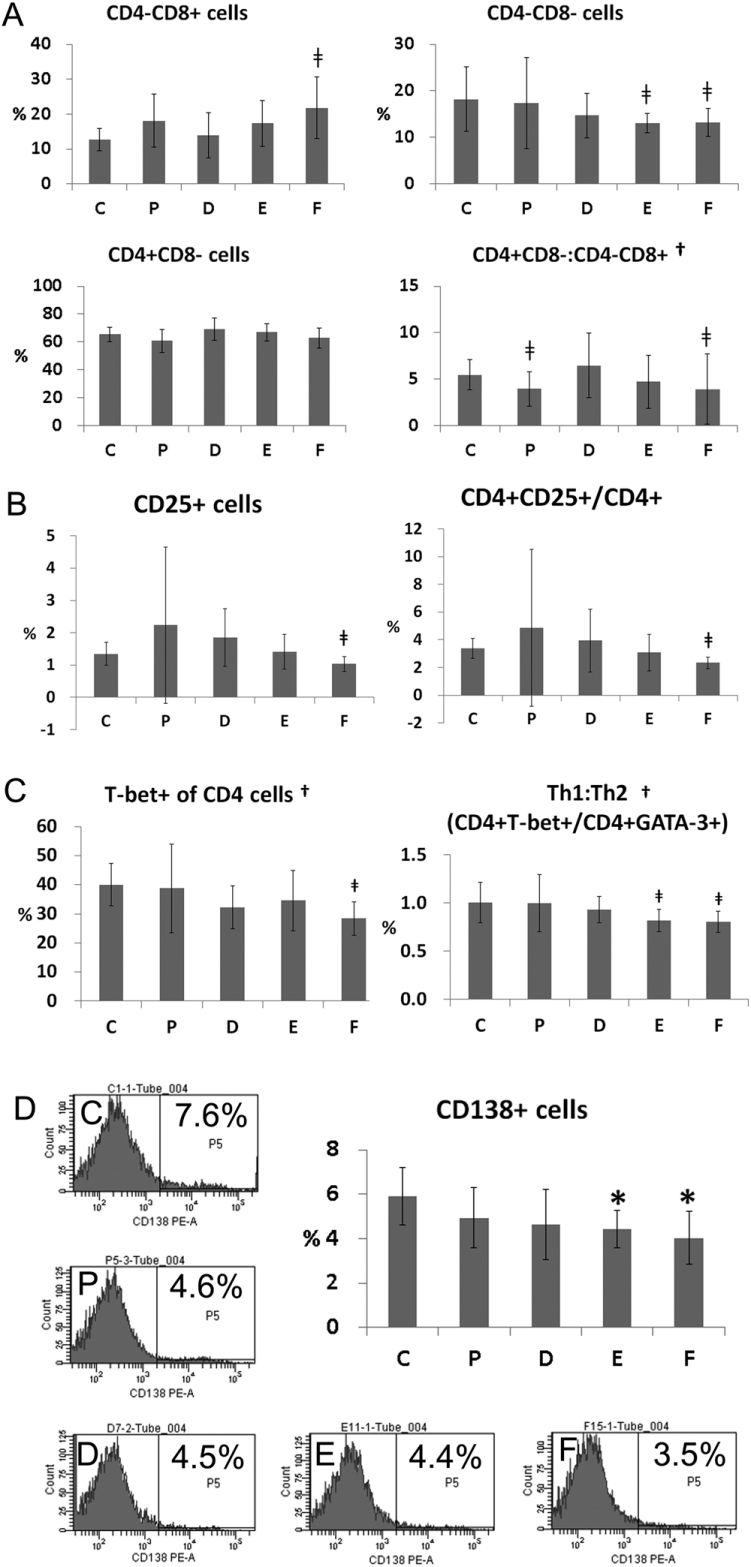
Flow cytometric determination of T cell, T helper cell subset, and plasma cell (CD138+ cells) proportions in the spleen. (**A**) Proportions of CD4^−^CD8^+^, CD4^−^CD8^−^, CD4^+^CD8^−^ cells and the ratio of CD4^+^CD8^−^ cells/CD4^−^CD8^+^ cells. Splenocytes were gated on CD3 and labeled with CD4 and CD8. (**B**) Proportion of CD25^+^ cells and CD4^+^CD25^+^/CD4^+^ cells. (**C**) proportion of T-bet^+^ CD4 cells and ratio of CD4^+^T-bet^+^ cells/CD4^+^GATA-3^+^ cells. Data obtained from each group were compared using the Kruskal Wallis test (^†^*p* < 0.05) followed by the Mann-Whitney U test^(†)^. Significant (p < 0.05) differences from the control (**C**) group are indicated by the symbol^‡^. (**D**) Proportion of CD138^+^ cells. Graphs and representative multi-color flow-cytometric gating analysis images from each group are presented. Data obtained from each group were compared using one-way analysis of variance (ANOVA) followed by *post hoc* Tukey’s multiple comparison tests. *Significant (p < 0.05) difference from the control (**C**) group. C: control group (vehicle (ethanol: Kolliphor® EL: saline = 1:1:8) 150 μl), P: methylprednisolone (5 mg/kg/day) treatment group, D: CKD-506, 10 mg/kg/day, E: CKD-506, 30 mg/kg/day, F: 50 mg/kg/day treatment group.

A representative gating scheme of the T cell subset and CD138^+^ cells in the spleen is presented in Supplementary Fig. 2. When comparing the expression of CD4 and CD8 by gating on CD3^+^, the ratio of CD4^+^CD8^−^/CD4^−^CD8^+^ T cells was significantly different (Kruskal-Wallis, *p* = 0.026, Fig. 4A). The ratio of CD4^+^CD8^−^/CD4^−^CD8^+^ T cells was significantly lower in groups methylprednisolone and CKD50 than group vehicle (Mann-Whitney U test, vehicle vs. P: *p* = 0.025; vehicle vs. CKD50: *p* = 0.003). There were significantly more CD4^−^CD8^+^ T cells in group CKD50 than in group vehicle (Mann-Whitney U test, vehicle vs. CKD50: *p* = 0.005, ANOVA & Tukey, *p* = 0.019).

There were significantly fewer CD4^−^CD8^−^ double-negative T cells in groups CKD30 and CKD50 than in group vehicle (Mann-Whitney U test, vehicle vs. CKD30: *p* = 0.01; vehicle vs. CKD50: *p* = 0.035, Fig. 4A). Compared with group vehicle, there were 4.3% fewer CD4^−^CD8^−^ cells in group methylprednisolone, 19.1% fewer in group CKD10, 28.3% fewer in group CKD30, and 27.3% fewer in group CKD50.

The proportions of CD4^+^CD25^+^Foxp3^+^, CD4^+^CD25^+^RORγt^+^, CD4^+^CD25^+^T-bet^+^, and CD4^+^CD25^+^GATA-3^+^ T cells in the spleen were analyzed to examine the expression levels of Foxp3, ROR-γt, T-bet, and GATA-3 as Treg, Th17, Th1, and Th2 master regulator cells, respectively. There were no statistically significant differences among groups (ANOVA & Tukey or Kruskal-Wallis). The percentage of CD25^+^ cells and CD4^+^CD25^+^/CD4^+^ ratio were significantly lower in group CKD50 than group vehicle (Mann-Whitney U test, vehicle vs. CKD50: *p* = 0.042 and *p* = 0.002, respectively, Fig. 4B).

T-bet expression by CD4^+^ cells was significantly different between groups (Kruskal-Wallis, P = 0.017), and T-bet expression by CD4^+^ cells in group CKD50 was significantly lower than in group vehicle (Mann-Whitney U test, *p* = 0.002, Fig. 4C). The ratio of CD4^+^T-bet^+^/CD4^+^GATA-3^+^ (expression ratio of Th1/Th2 master regulator CD4 cells) was also significantly different between groups (Kruskal-Wallis, *p* = 0.007), and the CD4^+^T-bet^+^/CD4^+^GATA-3^+^ ratios in groups CKD30 and CKD50 were significantly lower than that in group vehicle (Mann-Whitney U test, vehicle vs. CKD30: *p* = 0.013, vehicle vs. CKD50: *p* = 0.003, Fig. 4C).

### Proportion of CD138^+^ cells in the spleen

Analysis of CD138 expression in the spleen revealed that the proportions of CD138^+^ cells in groups CKD30 and CKD50 were significantly lower than in group vehicle (ANOVA & Tukey, *p* = 0.019, Fig. 4D). Compared with group vehicle, group methylprednisolone had 16.3% fewer CD138^+^ cells, group CKD10 21.2% fewer CD138^+^ cells, group CKD30 25.2% fewer CD138^+^ cells, and group CKD50 31.6% fewer CD138^+^ cells.

### Serum cytokines

Serum cytokines measured at the time of autopsy showed large standard deviations among individuals, with levels of cytokines below the detectable range in some individuals. Therefore, data were evaluated using nonparametric statistics (ranking method; see Table 1). Levels of IL-10 (*p* = 0.017), IL-12 (70) (*p* = 0.038), IL-15 (*p* = 0.001), IL-17 (*p* = 0.020), and TNF-α (*p* = 0.001) were significantly different among groups by the Kruskal-Wallis test.

**Table 1:** Serum cytokine levels in the various experimental and control groups.

pg/ml	C (n = 8)	P (n = 13)	D (n = 10)	E (n = 14)	F (n = 14)
GM-CSF	8.61 ± 16.51	ND	2.65 ± 8.38	5.32 ± 15.20	1.89 ± 7.09
IFN-γ	5.86 ± 12.51	0.07 ± 0.24	1.06 ± 3.34	6.60 ± 15.24	2.07 ± 5.38
IL-10^†^	1876.37 ± 5107.59	18.41 ± 12.97*	25.00 ± 25.33	56.55 ± 146.53	8.31 ± 14.15*
IL-12(70)^†^	2514.76 ± 6898.68	1.04 ± 3.76*	1.94 ± 3.69	74.50 ± 238.09	15.62 ± 45.25
IL-15^†^	255.59 ± 337.55	45.25 ± 14.91*	184.36 ± 280.13	162.41 ± 200.63	26.24 ± 28.73*
IL-17^†^	581.77 ± 1579.95	3.59 ± 1.73*	10.40 ± 12.20	21.14 ± 54.60	4.82 ± 7.15*
IL-1α	289.98 ± 337.54	666.21 ± 527.20	371.96 ± 261.58	458.40 ± 339.71	629.16 ± 480.71
IL-1β	1.58 ± 3.14	0.65 ± 2.34	1.27 ± 2.04	74.66 ± 264.02	3.86 ± 7.83
IL-2	6.96 ± 14.91	0.09 ± 0.34	1.91 ± 5.61	9.32 ± 17.79	4.09 ± 9.84
IL-4	51.52 ± 127.41	1.49 ± 0.20	1.74 ± 0.60	8.60 ± 25.44	3.70 ± 6.48
IL-6	1275.32 ± 3544.87	3.36 ± 3.30	7.21 ± 7.41	21.23 ± 50.94	5.69 ± 8.94
TNF-α^†^	7.33 ± 4.02	1.57 ± 2.07*	7.91 ± 8.45	16.25 ± 50.35	1.24 ± 2.60*
TGF-β1	51494.48 ± 23604.57	66067.78 ± 18777.09	73356.45 ± 22951.98	58807.36 ± 25015.67	73110.92 ± 17325.08*
IL-22	31.00 ± 46.45	17.68 ± 16.19	27.05 ± 31.48	13.00 ± 16.48	6.37 ± 5.56*
IL-23	126.59 ± 193.48	22.31 ± 61.77	1.96 ± 6.20	100.56 ± 376.25	41.34 ± 154.68

Data are expressed as mean ± SD. Data obtained from each group were compared using the Kruskal Wallis test (^†^*p* < 0.05) followed by the Mann-Whitney U test (*). *Significant (p < 0.05) differences compared with the control group (group C) are marked by an asterisk. C: control group (vehicle (ethanol: Kolliphor® EL: saline = 1:1:8) 150 μl), P: methylprednisolone (5 mg/kg/day) treatment group, D: CKD-506, 10 mg/kg/day, E: CKD-506, 30 mg/kg/day, F: 50 mg/kg/day treatment group, GM-CSF: granulocyte macrophage-colony stimulating factor, IFN-γ: interferon-γ, IL: interleukin, TGF-β1: transforming growth factor beta 1, SD: standard deviation.

The Mann-Whitney U test revealed that levels of IL-10 (*p* = 0.003), IL-15 (*p* = 0.002), IL-17 (*p* = 0.005), TNF-α (*p* = 0.003), and IL-22 (*p* = 0.008) were significantly lower in group CKD50 than in group vehicle, while levels of TGF-β1 (*p* = 0.035) were significantly higher in group CKD50 than in group vehicle. Levels of IL-10 (*p* = 0.025), IL-12 (70) (*p* = 0.038), IL-15 (*p* = 0.001), IL-17 (*p* = 0.02), and TNF-α (*p* = 0.001) were significantly lower in group methylprednisolone than group vehicle. There was no statistically significant difference in cytokine levels between groups CKD10 and vehicle or between groups CKD30 and vehicle.

### Serum levels of IP-10 and CCL2

**Figure 5:**
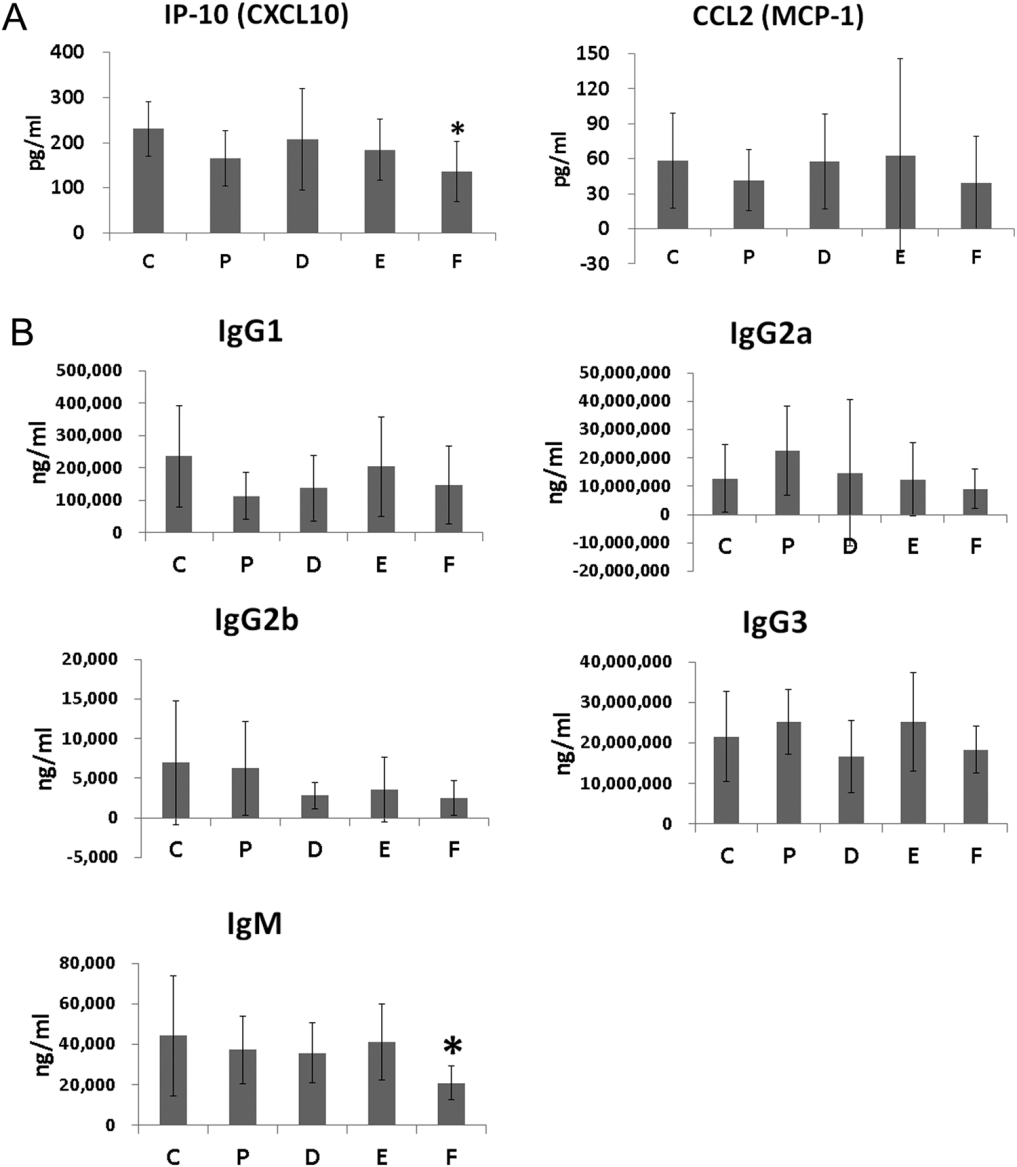
Serum concentrations of interferon-inducible protein 10 (IP-10), chemokine (C-C motif) ligand 2 (CCL2), and immunoglobulin isotypes. (**A**) Serum concentrations of IP-10 and CCL2 (**B**) immunoglobulin isotypes (IgG1, IgG2a, IgG2b, IgG3, and IgM). Data obtained from each group were compared using one-way analysis of variance (ANOVA) followed by *post hoc* Tukey’s multiple comparison tests. *Significant (p < 0.05) difference from the control (C) group. Ig: immunoglobulin. C: control group (vehicle (ethanol: Kolliphor® EL: saline = 1:1:8) 150 μl), P: methylprednisolone (5 mg/kg/day) treatment group, D: CKD-506, 10 mg/kg/day, E: CKD-506, 30 mg/kg/day, F: 50 mg/kg/day treatment group.

When serum levels of IP-10 and CCL2 were measured at 36 weeks of age, serum levels of IP-10 were found to be significantly lower in group CKD50 than group vehicle (ANOVA & Tukey, *p* = 0.016, Fig. 5A). Mean serum levels of CCL2 were lower in groups methylprednisolone and CKD50 groups than group vehicle but this difference was not statistically significant.

### Serum levels of immunoglobulins

Mean concentrations of IgG1, IgG2a, IgG2b, IgG3, and IgM were lower in group CKD50 than in group vehicle, and levels of IgM were significantly lower in group CKD50 than group vehicle (ANOVA & Tukey, *p* = 0.017, Fig. 5B).

### Cytokine levels in kidneys

Levels of various cytokines were measured in kidney extracts at the time of autopsy. Levels of IFN-γ, IL-1β, IL-4, IL-6, IP-10, MCP-1, CCL4, and RANTES were significantly different among groups based on ANOVA (Table 2).

**Table 2:** Levels of various cytokines and chemokines in kidney extracts.

pg/ml	C (n = 8)	P (n = 13)	D (n = 10)	E (n = 14)	F (n = 14)
IFN-γ^†^	25.73 ± 7.95	16.91 ± 5.10	17.46 ± 15.04	13.12 ± 4.85*	6.77 ± 4.06*
IL-10	10.06 ± 4.48	12.14 ± 2.02	9.43 ± 4.36	9.62 ± 2.42	7.60 ± 2.08
IL-15	36.27 ± 19.38	40.90 ± 9.01	34.25 ± 21.24	42.42 ± 16.89	32.82 ± 11.63
IL-1β^†^	4.63 ± 4.14	4.80 ± 2.85	2.09 ± 2.68	1.18 ± 2.19*	0.26 ± 0.60*
IL-4^†^	0.12 ± 0.17	0.06 ± 0.08	0.01 ± 0.02*	0.02 ± 0.05*	Not detected*
IL-6^†^	6.00 ± 4.76	2.95 ± 0.59*	3.99 ± 2.72	1.65 ± 0.82*	0.43 ± 0.45*
TNF-α	1.30 ± 2.45	0.07 ± 0.24	Not detected	Not detected	Not detected
IP-10^†^	101.99 ± 59.98	50.64 ± 15.63*	98.47 ± 44.61	63.40 ± 21.06*	43.90 ± 14.50*
CCL2^†^	107.99 ± 87.10	19.91 ± 11.71*	61.30 ± 70.39	21.90 ± 17.10*	3.15 ± 6.10*
CCL4^†^	14.14 ± 13.57	0.60 ± 1.84*	12.77 ± 23.90	1.70 ± 4.61	Not detected*
RANTES^†^	20.90 ± 20.92	9.77 ± 15.22	31.17 ± 24.93	9.30 ± 7.86	3.80 ± 2.17

Data are expressed as mean ± SD. Data obtained from each group were compared using one-way analysis of variance (ANOVA, ^†^p < 0.05) followed by *post hoc* Tukey’s multiple comparison tests. *Significant (p < 0.05) difference from the control (C) group. C: control group (vehicle (Ethanol: Kolliphor® EL: saline = 1:1:8) 150 μl), P: methylprednisolone (5 mg/kg/day) treatment group, D: CKD-506, 10 mg/kg/day, E: CKD-506, 30 mg/kg /day, F: 50 mg/kg/day treatment group, IFN-γ: interferon-γ, IL: interleukin, IP-10: IFN-inducible protein 10, CCL: chemokine (C-C motif) ligand, RANTES: regulated on activation, normal T cell expressed and secreted, SD: standard deviation.

*Post hoc* Tukey’s multiple comparison tests revealed that levels of IFN-γ, IL-1β, IL-4, IL-6, IP-10, MCP-1, and CCL4 were significantly lower in group CKD50 than in group vehicle. Levels of IFN-γ, IL-1β, IL-4, IL-6, IP-10, and MCP-1 were significantly lower in group CKD30 than group vehicle. Levels of IL-6, IP-10, MCP-1, and CCL4 were significantly lower in group methylprednisolone than in group vehicle. There were no statistically significant differences in cytokine expression between groups CKD10 and vehicle with the exception of IL-4.

### Serum chemistry and histopathology

Serum levels of ALT (*p* = 0.001), ALP (*p* = 0.001), albumin (*p* = 0.001), and total protein (*p* = 0.018) were significantly different among groups (ANOVA). In multiple comparisons, ALT was significantly higher in group CKD50 than the other groups (Tukey), but still within the reference range. Compared with groups vehicle and CKD10, albumin was significantly higher in group methylprednisolone (Tukey). Table 3 shows the results of parametric statistical analysis. Necropsy was conducted on all animals at the end of experiment, and administration of CKD506 did not result in significant changes in appearance of the spleen, kidneys, liver, lungs, or heart based on gross examination or microscopic examination.

**Table 3:** Serum chemistry data from experimental and control groups.

Serum chemistry parameters	C	P	D	E	F
ALT (U/l)^†^	15.25 ± 4.92	24.09 ± 6.64	18.10 ± 5.43	20.79 ± 2.91	29.07 ± 12.21*
AST (U/l)	39.75 ± 20.10	52.09 ± 14.10	44.20 ± 10.09	55.21 ± 19.34	59.00 ± 56.14
ALP (U/l)^†^	186.38 ± 40.29	207.45 ± 23.90	187.44 ± 62.24	155.92 ± 19.94	153.08 ± 17.25
Total bilirubin (mg/dl)	0.25 ± 0.21	0.41 ± 0.29	0.22 ± 0.09	0.26 ± 0.12	0.28 ± 0.14
Albumin (g/dl)^†^	1.89 ± 0.38	2.52 ± 0.25*	1.91 ± 0.43	2.19 ± 0.43	2.26 ± 0.35
Total protein (g/dl)^†^	4.71 ± 0.58	5.42 ± 0.39	4.74 ± 0.63	5.21 ± 0.71	5.35 ± 0.60
CPK (U/l)	99.38 ± 90.59	64.36 ± 14.14	86.70 ± 56.89	84.71 ± 31.40	125.86 ± 177.18
Ca (mg/dl)	10.10 ± 1.70	9.35 ± 0.53	10.08 ± 1.26	9.65 ± 0.69	9.43 ± 0.59

Data are expressed as mean ± SD. Data obtained from each group were compared using one-way analysis of variance (ANOVA, ^†^p < 0.05) followed by *post hoc* Tukey’s multiple comparison tests. *Significant (p < 0.05) difference from the control (C) group. C: control group (vehicle (ethanol: Kolliphor® EL: saline = 1:1:8) 150 μl), P: methylprednisolone (5 mg/kg/day) treatment group, D: CKD-506, 10 mg/kg/day, E: CKD-506, 30 mg/kg /day, F: 50 mg/kg/day treatment group, ALT: alanine aminotransferase, AST: aspartate aminotransferase, ALP: alkaline phosphatase, CPK: creatine phosphokinase, SD: standard deviation.

## Discussion

SLE has a significant unmet medical need because there are very limited treatment options currently available for this devastating disease. In this study, we analyzed the therapeutic potential of a novel hydroxamate HDAC6-selective inhibitor, CKD-506.

A phase I clinical trial for CKD-506 was completed and did not reveal significant safety issues (EudraCT number: 2016-002816-42). Previous research supports the safety of HDAC6 inhibitors. First, HDAC6 knockout mice develop normally without any significant abnormalities^17^. Second, HDAC6 expression is induced under stressful pathologic conditions^18^, such that HDAC6 selective inhibitors are usually safe under normal conditions.

CKD-506 inhibited HDAC6 very selectively in enzyme activity assays and induced acetylation of α tubulin. In human PBMC, CKD-506 induced tubulin acetylation in a dose-dependent manner without affecting histone acetylation. In mouse, CKD-506 showed dose-dependent increase in plasma exposure. In previous studies, overexpression of HDAC6 significantly increased expression of pro-inflammatory cytokines with concomitant reduction in acetylated α-tubulin^16^, and LPS-induced production of pro- and anti-inflammatory cytokines has been affected by inhibition of HDAC6 in macrophages^19,20^. Thus, the immunomodulatory properties of HDAC6 inhibitor promoted us to assess the efficacy of CKD506 in SLE. Macrophage activation plays a critical role in inflammation and is a component of both innate and adaptive immune systems, which are both dysregulated in SLE.

Several HDAC6 selective inhibitors such as Tubastatin A and ACY-1215 showed excellent immunosuppressive activity in animal studies; they prevented the development of contact hypersensitivity and experimental graft-versus-host disease-like diseases and improved long-term survival in a lethal septic model^13,21^. HDAC 6 inhibitors reduced synovial inflammation and joint destruction in a mouse model of collagen antibody-induced arthritis^22^ and pathogenesis in murine SLE^23^.

Although tricyclic Tubastatin A has good HDAC6 inhibitory activity and isotype selectivity, it has shown limited success in clinical trials because of its poor pharmacokinetic properties and potential genotoxicity^24–26^. Another HDAC6 inhibitor, ACY-1215, which is in a phase 2 clinical trial for multiple myeloma, has high HDAC6 enzyme potency but low isotype selectivity (11-fold selectivity for HDAC6 and HDAC1 at IC_50_)^27^. Due to its low isotype selectivity, significant adverse effects such as grade 3 anemia and neutropenia have been reported in clinical trials^28,29^.

In contrast, CKD-506 showed excellent isotype selectivity (>100-fold over other HDAC family members).

When NZB/W F1 mice had been treated with CKD-506, survival rate was significantly prolonged and the incidence of severe proteinuria was significantly reduced.

CD138 is expressed by B cell precursors and plasma cells, and plasma cells generated during autoimmune responses are responsible for autoantibody production^30^. It was also reported that the serum level of sCD138, the expression of CD138 mRNA in PBMC, and the numbers of CD20^−^ CD38^+^CD138^+^ plasma cells were increased in patients with active SLE in comparison with normal controls^31^. Thus, the reduction of CD138^+^ cells by CKD-506 may have contributed to the reduction of anti-dsDNA antibody and immune complexes, resulting in improvement in histopathological features of the kidney and symptoms of disease.

A previous study reported that the proportion of CD4^−^CD8^−^ double negative T cells was significantly higher in PBMCs from SLE patients, and these cells have been shown to contribute to the pathogenesis of renal injury in SLE by producing the inflammatory cytokines IL-17 and IFN-γ^32,33^. In this study, CKD-506 treatment significantly decreased the ratio of CD4^−^CD8^−^ double negative T cells and serum level of IL-17 in a murine model of SLE, which also may have contributed to prevention of the pathogenesis of renal injury.

In a human study, serum levels of IL-6, IL-10, IL-17, IL-23, and IFN-γ were significantly higher and serum levels of TGF-β were significantly lower in SLE patients than in healthy controls^34^.

CKD-506 treatment significantly reduced serum levels of IL-10, IL-15, IL-17A, TNF-α, and IL-22 and significantly increased serum level of TGF-β1 in a murine model of SLE.

IL-10 has both anti-inflammatory and immune stimulatory effects; it promotes B cell proliferation and Ig class switching in addition to increasing autoantibody production in SLE^35^.

In a previous study, serum IL-15 levels were found to be increased in approximately 38% of SLE patients, but not in healthy controls^36^. IL-15 stimulates T cells and has also been shown to stimulate B cell proliferation and differentiation^37^. Because inappropriate immune regulation, excessive autoantibody production, and hyperactive B cells are characteristic of SLE^38^, the decrease in IL-15 concentration we observed may have been associated with the CKD-506-induced mitigation of SLE in our mouse model.

TNF-α levels have been shown to be increased in SLE patients, and to correlate with disease activity^39^. Immune complexes induce the production of TNF-α from monocytes^40^, and TNF-α has its own proinflammatory activity and causes vascular injury and organ damage^41^.

Many studies have suggested that IL-17 is involved in the pathogenesis of SLE; the number of IL-17-producing cells, including CD4^+^Th17 cells and double negative T cells, has been shown to be elevated in peripheral blood from SLE patients^33^, and IL-17 levels have been shown to be increased in the sera of SLE patients^42^.

In addition to IL-17A, Th17 cells produce IL-17F, IL-21, and IL-22^43^. IL-17 exerts proinflammatory effects by stimulating the production of IL-6, GM-CSF, and G-CSF^44^ and promoting the recruitment of monocytes and neutrophils, resulting in tissue inflammation and damage^43^.

TGF-β promotes the production of Treg cells by inducing Foxp3 expression^45^, while stimulating Th17 differentiation in an IL-6 inflammatory setting^46^. In SLE, Th17 and Treg cells are in balance, and this balance can change according to disease activity^47^.

Serum TGF-β1 levels were significantly higher in group CKD50, but no increase in the number of Treg cells (CD4^+^CD25^+^Foxp3^+^) in the spleen was observed. Rather, the expression of CD25^+^ cells and the ratio of CD4^+^CD25^+^/CD4^+^ cells were significantly lower in group CKD50 than group vehicle, suggesting that T cell activation was reduced by CKD-506, because CD25 is expressed on the surface of activated T cells. Activated SLE T cells play a role in the production of autoantibodies by B cells, so the reduction in T cell activation may have contributed to the decrease in the disease activity of SLE. In previous studies on Treg, genetic targeting of HDAC6 (HDAC6^−/−^) did not affect Treg numbers or Foxp3 expression but affected the phenotype of Treg and increased Treg suppressive function^48^. In addition, heat shock response had an important role in the improvement of Treg suppressive function mediated by HDAC6 inhibition^49^.

Th1 and Th2 master regulator analysis of CD4^+^ cells revealed that expression of T-bet, which is a Th1 master regulator, and the ratio of Th1/Th2 master regulator CD4 cells were significantly decreased by CKD-506. Previous studies on the Th1/Th2 balance in the peripheral blood of SLE patients showed a high proportion of Th1 cells in SLE patients with WHO class IV glomerulonephritis^50^. Therefore, a reduction in T-bet expression and the Th1/Th2 ratio may have contributed to the improvement in SLE disease characteristics in this study.

Levels of IP-10 were significantly decreased by CKD-506. IP-10 is a chemokine secreted from cells stimulated with type I and II interferons and LPS, and specifically induces Th1 cells^51,52^.

Recently, IFN-α has been implicated as an important cytokine in SLE^53,54^, and its response protein, IP-10, has also been studied. Several studies of SLE patients have shown that SLE activity is highly correlated with serum level of IP-10^52,55,56^. A previous study suggested that IP-10 is biomarker of disease activity in SLE^54^. The specificity of IFN- α ELISAs has been questioned^57^, while indirect assessment of IFN activity has been reported to be much more sensitive^58^. IP-10 is ligand of CXCR3 that recruit activated CD4^+^ and CD8^+^ T cells^59,60^.

We found that CKD-506 significantly decreased levels of IFN-γ, IL-1β, IL-4, IL-6, IP-10, MCP-1, and CCL4 in the kidney extracts of mice with lupus nephritis. IFN-γ, IL-1, IL-4, IL-6, 1L-10, and TNF-α have previously been reported to be expressed in the kidneys of animal models with lupus nephritis^61,62^. Furthermore, the chemokines CCL2 (MCP-1), CCL4 (MIP-1β), RANTES (CCL5), and IP-10 (CCL10) have been found to be expressed in the kidneys of murine models of SLE^63–66^. Consistent with data from these animal studies, the cytokines and chemokines listed above have been detected in kidney biopsies from SLE patients^66–69^. Furthermore, a previous study of human patients reported that CXCR3^+^CD4^+^ T cells recruited by IP-10 are valuable markers of disease activity in lupus nephritis^70^. In this study, CKD-506 decreased the expression of IP-10.

HDAC6 activates the NADPH oxidase-reactive oxygen species (ROS)-NFkB pathway that results in the production of a significant amount of inflammatory cytokines such as TNF-α and type 1 IFNs^16^. CKD-506 also repressed IFN promoter activity in the THP-1 blue IGS monocyte cell line (Supplementary Fig. 3), and CKD-506 repressed TNF-α expression significantly in the blood of NZB/W F1 mice, suggesting that this signaling pathway may be involved in the efficacy of CKD-506. Thus, CKD-506 may reduce the pathogenesis of SLE by repressing innate immune systems such as production of TNF-α and type1 interferons.

The plasma concentration of CKD-506 was above 200 nM (84.5 ng/ml) for at least 4 hr in 50 mg/kg treated group and for at least 2 hr in 30 mg/kg treated group. When considering IC_90_ of CKD-506 for both HDAC6 enzyme activity and the induction of α tubulin acetylation is around 100 nM, 50 mg/kg of CKD-506 is enough to inhibit *in vivo* HDAC6 activity for more than 4 hr and 30 mg/kg of CKD-506 is enough for more than 2 hr. Because 30 mg/kg of CKD-506 once a day administration showed significant therapeutic efficacy, even brief inhibition of HDAC6 may provide prolonged therapeutic efficacy *in vivo*.

In summary, we demonstrated that CKD-506 suppressed lupus nephritis without significant adverse effects by repressing the expression of lupus disease-specific cytokines and chemokines in serum and kidneys and, as a result, the migration of inflammatory cells. These results suggest the possibility of use of CKD-506 for treatment of SLE.

## Materials and Methods

### Chemicals

CKD-506 is a new histone deacetylase 6 inhibitor developed by the Chong Kun Dang Pharmaceutical Corporation (CKD Pharm, Korea). LBH589, pan HDAC inhibitor, was purchased form Lc Labs, USA (P-3703). For the *in vitro* enzyme assay and tubulin acetylation assay, these chemicals were dissolved in dimethyl sulfoxide (DMSO; Sigma-Aldrich, St. Louis, MO, USA). The stock solutions had been kept at −20 °C and were diluted to the required concentrations in growth media as necessary. Vehicle (DMSO) was used in the negative control groups. The final concentration of DMSO was less than 0.1%.

### Enzyme assay

HDAC panel assay was performed by Reaction biology Corp. (Malvern, PA, USA) according to their validated protocol. To evaluate the potency and selectivity of CKD-506, HDAC panel assay was carried out by Reaction biology Corp. Briefly, the deacetylation reaction was performed in 50 mM Tris-HCl pH 8.0, 137 mM NaCl, 2.7 mM KCl, 1 mM MgCl_2_, and 1 mg/ml bovine serum albumin (BSA) with RHK-K(Ac)-AMC as substrate. After the reaction, a fluorescence signal (Ex. 360 nm/Em. 460 nm) developed (~30 min.) by addition of an equal volume of 2 mM nicotinamide, 16 mg/mL trypsin in 50 mM Tris-HCl pH 8.0, 137 mM NaCl, 2.7 mM KCl, and 1 mM MgCl_2_.

### The analysis of protein acetylation

Human peripheral blood mononuclear cells (PBMC) (IRB No. CKD-IRB-012) were seeded (1.0 × 10^6^ cells/well) and cultured for 24 hr on 12-well plate before treatment of CKD-506 or LBH-589. The chemicals were treated for 4 hr. After harvesting cells, total protein extracts were prepared. The protein extracts were resolved in pre-made SDS-PAGE (NuPAGE® Bis-Tris Precast Gels, Invitrogen). Proteins were transferred onto polyvinylidene difluoride (PVDF) membrane and probed with the following antibodies; anti-acetyl α tubulin (1:5000, #5335, Cell Signaling Technology), α tubulin (1:2000, #2144, Cell Signaling Technology), anti-acetyl histone H4(1:5000, #8647, Cell Signaling Technology), anti-histone H4(1:1000, #2935S, Cell Signaling Technology), and anti-beta-actin (1:5000, #A5441, Sigma). The horseradish peroxidase-conjugated anti-rabbit IgG (1:5000, #7074S, Cell Signaling Technology) and anti-mouse IgG (1:5000, #7076 S, Cell Signaling Technology) were used as secondary antibodies. Acetylated proteins were visualized by chemiluminescence (RPN2235, GE healthcare) and detected by the Gel documentation system (ChemiDoc™, BioRad).

### Pharmacokinetic analysis of CKD-506

Four 23-week old female NZB/W F1 mice were purchased from Jackson Laboratory and housed in CKD Research Institute animal facility. This study was reviewed and approved by the Institutional Animal Care and Use Committee of CKD Research Institute. All procedures were in compliance with Animal Welfare Act Regulations and the Guide for the Care and Use of Laboratory Animals. The indicated amount of CKD-506 was injected intraperitoneally into NZB/W F1 mice. Blood samples were collected from retro-orbital plexus using a heparinized capillary tube under diethyl-ether anesthesia at pre-dose (0) and 0.25, 1, 2, 4, 8 and 24 hours post-dose at day of PK sampling. After centrifugation at 13,000 rpm for 4 min and 4 °C, ten microliters of the separated plasma was dispensed into microtube and 120 μl of organic solvent (90% acetonitrile (in MeOH, v/v)) was immediately added for deproteinization/extraction. The pre-treated samples had been kept at −70 °C until analysis. Ten microliters of internal standard (verapamil 500 ng/ml in acetonitrile) was added into the pre-treated plasma samples and vortexed for 1 min and then, centrifuged at 13,000 rpm for 4 min and 4 °C. Eighty microliters of the supernatant of the sample were transferred into a 96 well-autosampler plate and 40 μl of HPLC water was then added in each well. The concentration of CKD-506 in samples were analyzed using API 4000 QTRAP (AB SCIEX) (Framingham, MA).

### Experimental animals and groups

Eighty female NZB/W F1 mice aged 4 weeks were purchased from Jackson Laboratory (Bar Harbor, ME, USA) and housed in SPF room of Samsung Biomedical Research Institute. The animals had been fed ad libitum throughout the study. This study was reviewed and approved by the Institutional Animal Care and Use Committee of Samsung Biomedical Research Institute. Samsung Biomedical Research Institute is accredited by the Association for the Assessment and Accreditation of Laboratory Animal Care International and abides by the guidelines of the Institute of Laboratory Animal Resources. All procedures were in compliance with Animal Welfare Act Regulations and the Guide for the Care and Use of Laboratory Animals.

Body weight and urinary protein level were measured at 22 and 24 weeks of age (immediately before initiation of treatment). Mice with a urinary protein concentration greater than 100 mg/dl were excluded from this study. Remaining subjects were divided into five groups containing 15 mice per group with an even distribution of urinary protein concentration and body weight among groups. Body weights (mean ± SD) at 24 weeks of age in the vehicle, methylprednisolone, CKD10, CKD30, and CKD50 groups were 42.7 ± 5.3 g, 42.7 ± 4.6 g, 42.6 ± 4.1 g, 42.6 ± 3.9 g, and 42.7 ± 5.2 g, respectively. Protein concentrations in the vehicle, methylprednisolone, CKD10, CKD30, and CKD50 groups were 41.6 ± 13.6 mg/dl, 49.2 ± 22.3 mg/dl, 43.8 ± 27.1 mg/dl, 40.6 ± 21.3 mg/dl, and 48.8 ± 15.2 mg/dl, respectively.

For the *in vivo* study, experimental groups consisted of a vehicle-treated control group (group vehicle, n = 15, negative treatment control), a methylprednisolone 5 mg/kg/day group (group methylprednisolone, n = 15, positive treatment control), a CKD-506 10 mg/kg/day group (group CKD10, n = 15), a CKD-506 30 mg/kg/day group (group CKD30, n = 15), and a CKD-506 50 mg/kg/day group (group CKD50, n = 15). CKD-506 was dissolved in vehicle (ethanol: Kolliphor® EL (Sigma Aldrich C5135, St. Louis, MO, USA): saline = 1:1:8) for injection. Methylprednisolone or CKD-506 was injected intraperitoneally from the age of 24 weeks until autopsy (43–44 weeks of age). Control mice received 150 μL of vehicle (Ethanol: Kolliphor® EL: saline = 1:1:8) and were subjected to the same schedule. Survival rate and incidence of severe proteinuria (percent individual mice with urine protein ≥300 mg/dL) were calculated. Survival had been monitored until 42 weeks of age.

### Determination of proteinuria

During the experiments, urine protein levels had been measured every 2 weeks. Fresh urine was collected by performing abdominal massages. Urine protein was measured with the Coomassie Brilliant Blue method as described in our previous study^71^. Urine creatinine was measured using a DRI-CHEM 3000 Colorimetric analyzer (Fujifilm, Tokyo, Japan) with the urine diluted in deionized water (1:100 dilution).

### Determination of blood urea nitrogen (BUN), serum creatinine-, and anti-dsDNA antibody levels

Blood samples had been collected from mice under isoflurane anesthesia every 4 weeks (at 24, 28, 32, 36, 40 weeks of age and autopsy), and the obtained serum samples had been kept at −70 °C until the analysis. Blood urea nitrogen (BUN) and serum creatinine levels were measured with a DRI-CHEM 3000 Colorimetric analyzer (Fujifilm, Tokyo, Japan) and anti-dsDNA antibody levels were measured using a mouse anti-dsDNA ELISA kit (Shibayagi Co. Ltd., Ishihara, Shibukawa, Japan).

### Hematoxylin and eosin (H&E), periodic acid/Schiff (PAS) reagent, and Masson trichrome staining

Kidneys were harvested from mice at the end of the study (at 43–44 weeks of age). For light microscopy, the tissues were fixed in 10% neutral buffered formalin, embedded in paraffin wax, sectioned at a thickness of 5 μm and stained with H&E (BBC Biochemical, Mount Vernon, WA, USA). Periodic acid/Schiff (PAS stain kit, BBC Biochemical) staining and Masson trichrome (BBC Biochemical) staining were used for the detection of proteinuria and fibrosis, respectively. Inflammatory cell infiltration and mesangial proliferation were scored on a 4-point scale as 0 (none), 1+ (mild), 2+ (moderate), 3+ (moderate to severe) and 4+ (severe).

### Immunofluorescence analysis of kidneys

Immunofluorescent staining was conducted and the fluorescence intensity of IgG and C3 deposits in the kidneys was evaluated as described previously using an LSM 700 laser-scanning confocal microscope (Carl Zeiss, Jena, Germany)^72^. Fluorescence staining intensities of IgG or C3 deposits were graded as 0 (none), 1+ (mild), 2+ (moderate), 3+ (moderate to strong) and 4+ (strong). Histopathology, IgG and C3 infiltration were evaluated in a blinded manner.

### Flow cytometry

Single cell suspensions were obtained from the spleens of NZB/W F1 mice at autopsy (at 43–44 weeks of age). The splenocytes were stained with PerCP-Cy5.5-conjugated anti-mouse CD3e (PerCP-cy5.5-CD3e, eBioscience, San Diego, CA, USA), fluorescein isothiocyanate (FITC)-conjugated anti-mouse CD4 (FITC-CD4, BD Biosciences, San Jose, CA, USA), PE-cyanine7-conjugated anti-mouse CD8a (eBioscience), allo-phycocyanin (APC)-conjugated anti-mouse CD25 (BD Biosciences) and PE-conjugated anti-mouse CD138 (BD Biosciences).

T cell profile was analyzed as described previously^2^; briefly, we examined proportions of Th1 cells (CD4^+^CD25^+^T-bet^+^), Th2 cells (CD4^+^CD25^+^GATA-3^+^), Th17 cells (CD4^+^CD25^+^ROR-γt^+^), and Treg cells (CD4^+^CD25^+^Foxp3^+^). To analyze T helper subsets, the splenocytes were stained with antibodies to CD4 and CD25 (FITC-conjugated anti-mouse CD4 and APC-conjugated anti-mouse CD25, BD Biosciences). Cells were fixed and permeabilized prior to staining with T-bet, GATA-3, ROR-γt and Foxp3 antibodies (PE-, BD Biosciences).

### Determination of serum cytokine levels

Serum samples were assayed using Milliplex® MAP Kits (Millipore) for granulocyte macrophage-colony stimulating factor (GM-CSF), interferon-γ (IFN-γ), interleukin-1α (IL-1α), IL-1β, IL-10, IL-12(p70), IL-15, IL-17a, IL-2, IL-4, IL-6, tumor necrosis factor α (TNF-α), transforming growth factor beta 1 (TGF-β1), IL-22, IL-23, IFN-inducible protein 10 (IP-10 (CXCL10)) and chemokine (C-C motif) ligand 2 (CCL2 (MCP-1)).

### Serum immunoglobulin isotyping

Serum immunoglobulin concentrations were determined with mouse IgG1, IgG2A, IgG2B, IgG3, and IgM Isotyping kits and a mouse Mag Isotyping assay base kit (R&D Systems, Minneapolis, MN). Thirty-fold diluted sera were used for analysis of IgM and IgG2B levels, 6000-fold diluted sera were used for analysis of IgG1 and IgG2A levels and 48,000-fold diluted sera were used for analysis of IgG3 level.

### Determination of cytokine levels in kidney extracts

The kidneys were weighed and then transferred into dPBS (Welgene) supplemented with anti-protease Complete TM cocktail (Boehringer) using 12.5 μl of dPBS per milligram of wet weight tissue. Samples were homogenized twice using an Automill TK-AM5 freeze-crushing cell homogenizer (W 280 mm × D 390 mm × H 290 mm, Tokken Inc., Chiba, Japan) at 1,400 rpm for 90 seconds and then centrifuged at 16,000 × *g* for 15 min at 4 °C. The supernatants were collected and total protein concentrations were determined in the kidney extracts using a bicinchoninic acid (BCA) protein assay kit™ (Pierce). Total protein concentrations of the kidney extracts were adjusted to 2 mg/dl and used for the analysis of IFN-γ, IL-10, IL-15, IL-17, IL-1β, IL-4, IL-6, IP-10, MCP-1, CCL4, RANTES, TNF-α and sCD40L with Milliplex® MAP Kits (Millipore).

### Serum chemistry

To determine if there were any changes in organ function due to long-term drug administration of the novel HDAC6 selective inhibitor, alanine aminotransferase (ALT), aspartate transaminase (AST), alkaline phosphatase (ALP), total protein, albumin, creatinine phosphokinase (CPK), total bilirubin, and calcium (Ca) levels were measured in the sera obtained at autopsy with a DRI-CHEM 3000 Colorimetric analyzer (Fujifilm, Tokyo, Japan).

### Statistical analysis

All results except for proteinuria and survival rate data are expressed as mean ± SD. Proteinuria and survival rate data were analyzed using Kaplan–Meier curves and the log-rank test. Serum cytokine levels and proportions of T cell subsets were compared using the Kruskal-Wallis test followed by the Mann-Whitney U test. Other data were compared among groups using one-way analysis of variance (ANOVA) followed by *post hoc* Tukey’s multiple-comparison tests. Two-group comparisons were performed with Student’s *t*-test. A *p*-value < 0.05 was considered statistically significant. All statistical analyses were conducted using SPSS version 22.0 (IBM, Armonk, NY, USA).

## Electronic supplementary material

Supplementary Information
