# Supplementary material for: CKD-506, a novel HDAC6-selective inhibitor, improves renal outcomes and survival in a mouse model of systemic lupus erythematosus
Source: Sci Rep. 2018 Nov 23;8:17297. doi: 10.1038/s41598-018-35602-1 (PMC6251916; doi:10.1038/s41598-018-35602-1)
Supplement: Supplementary file 1 — Supplementary Information [file 41598_2018_35602_MOESM1_ESM.pdf]

Supplementary Information

Title:

CKD-506, a novel HDAC6-selective inhibitor, improves renal outcomes and survival in a mouse model of systemic lupus erythematosus

Authors:

Eun Wha Choi[1,*], Ji Woo Song[2], Nina Ha[3], Young Il Choi[3], Sungjoo Kim[2,4,*]

[1]Department of Veterinary Clinical Pathology, College of Veterinary Medicine & Institute of Veterinary Science, Kangwon National University, 1 Kangwondaehak-gil, Chuncheon, Gangwon-do, 24341, Republic of Korea

[2]Transplantation Research Center, Samsung Biomedical Research Institute, Samsung Medical Center, 81 Irwon-ro, Gangnam-gu, Seoul, 06351, Republic of Korea

[3]CKD Research Institute, 315-20 Dongbaek Jukjeon-Daero, Yongin, 16995, Republic of Korea

[4]Department of Surgery, Division of Transplantation, Samsung Medical Center, Sungkyunkwan University School of Medicine, 81 Irwon-ro, Gangnam-gu, Seoul, 06351, Republic of Korea

*Corresponding author: Eun Wha Choi, DVM, PhD, Assistant Professor

Address: Department of Veterinary Clinical Pathology, College of Veterinary Medicine, Kangwon National University, 1 Kangwondaehak-gil, Chuncheon-si, Gangwon-do 24341, Republic of Korea

Telephone: 82-33-250-8794, Fax: 82-33-259-5625

E-mail: ewchoi@kangwon.ac.kr

*Corresponding author: SungJoo Kim, MD, PhD, Professor

Department of Surgery, Division of Transplantation, Samsung Medical Center, Sungkyunkwan University School of Medicine, 81 Irwon-ro, Gangnam-gu, Seoul 135-710, Republic of Korea

Telephone: 82-2-3410-3476

Fax: 82-2-3410-0040

E-mail: kmhyj111@gmail.com

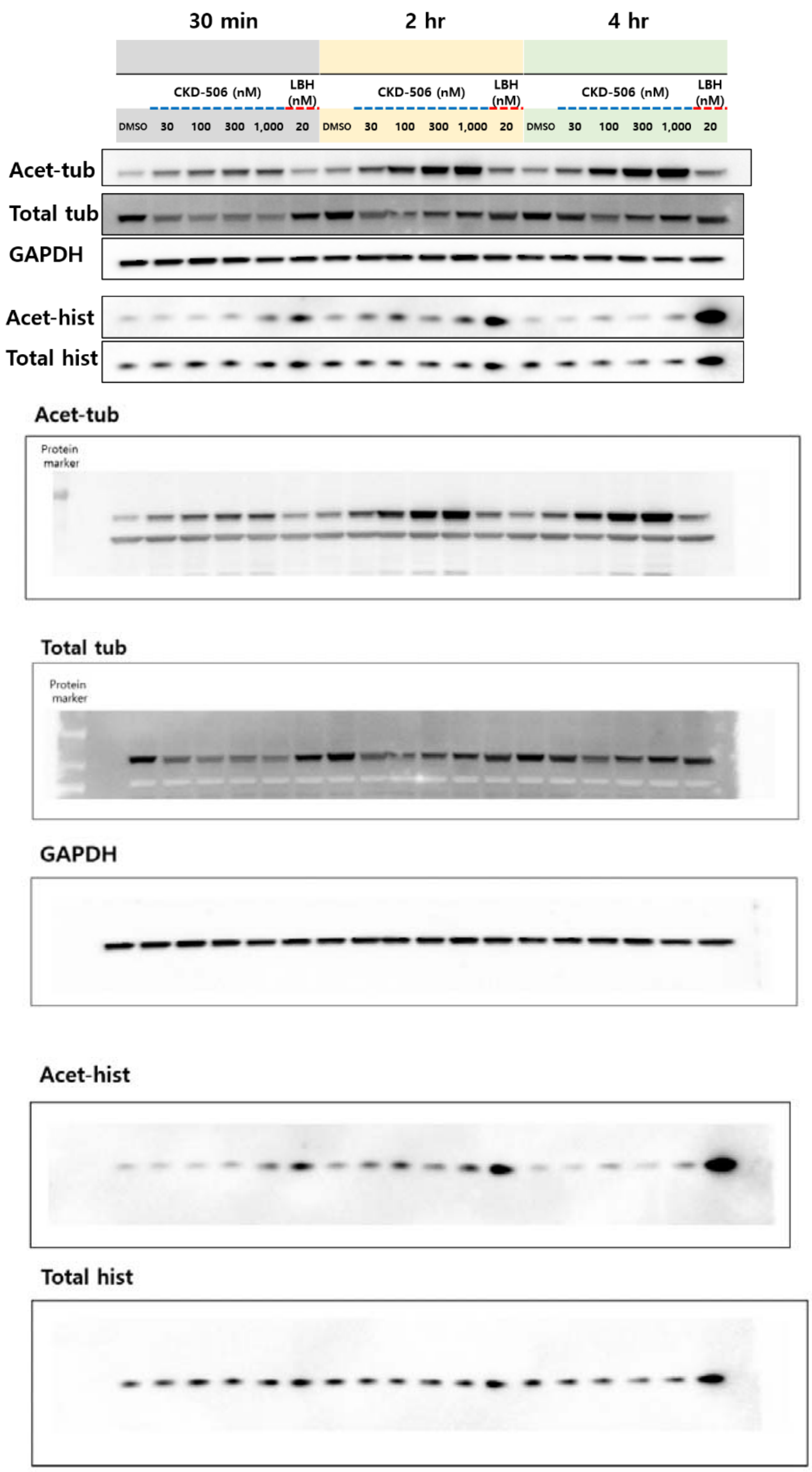

Acetylated α tubulin

α Tubulin

Acetylated Histone H4

Histone H4

Supplementary Figure 1. The selectivity of CKD-506 in human PBMC was analyzed based on the acetylation of α tubulin, one of the major target proteins of HDAC6, and the acetylation of histone H4 which is not a target protein of HDAC6. LBH-589 is a pan HDAC inhibitor, a positive control for the induction of histone H4 acetylation (30 min, 2hr and 4hr: the chemicals were treated for 30 min, 2hr and 4 hr, respectively.).

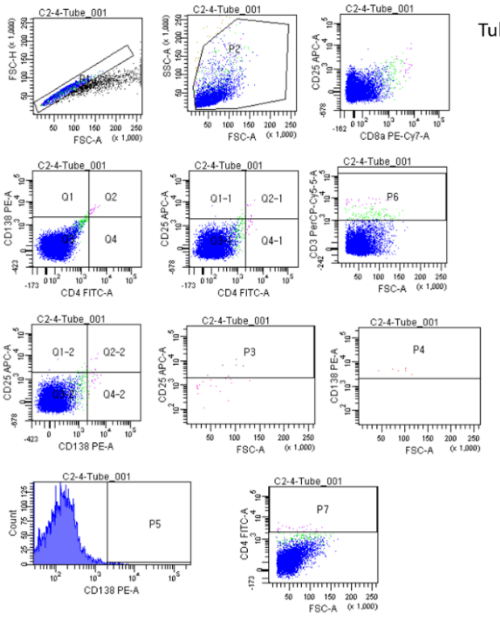

Tube 001: Unstained control tube

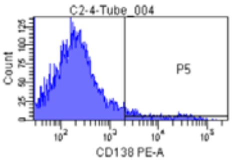

Tube 004:
CD138$^+$ cells (%)=P5 (%)

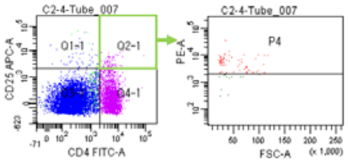

Tube 007:
CD4$^+$CD25$^+$Foxp3$^+$ (%)
=Q2-1 (%)× P4 (%)

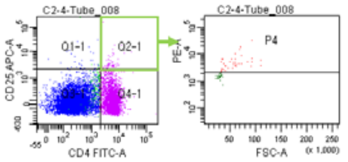

Tube 008:
CD4$^+$CD25$^+$RORγt$^+$ (%)
=Q2-1 (%)× P4 (%)

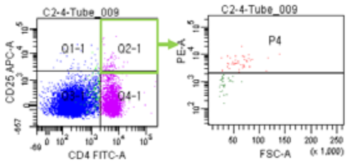

Tube 009:
CD4$^+$CD25$^+$T-bet$^+$ (%)
=Q2-1 (%)× P4 (%)

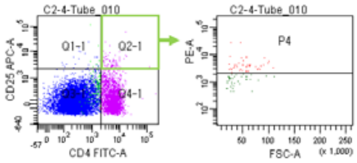

Tube 010:
CD4$^+$CD25$^+$GATA-3$^+$ (%)
=Q2-1 (%)× P4 (%)

Tube 007~011:
CD25$^+$ (%)=Q1-1+Q2-1 (%)
CD4$^+$CD25$^+$/CD4$^+$ (%)
=Q2-1 (%)/(Q2-1 (%)+Q4-1 (%))

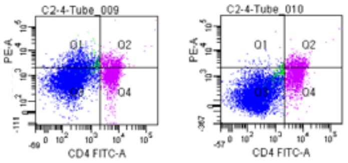

CD4$^+$T-bet$^+$/CD4$^+$GATA-3$^+$
=Q2 (%) of tube 009/Q2 (%) of tube 010

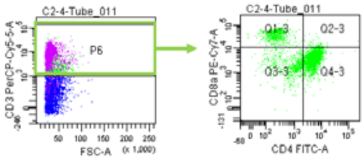

Tube 011:
CD4$^-$CD8$^+$ (%)=Q1-3 (%)
CD4$^-$CD8$^-$ (%)=Q3-3 (%)
CD4$^+$CD8$^-$ (%)=Q4-3 (%)
CD4$^+$CD8$^-$:CD4$^-$CD8$^+$
=Q4-3/Q1-3

Supplementary Figure 2. A representative gating scheme of the T cell subset and CD138^{+} cells in the spleen.

To analyze T helper subsets, splenocytes were stained with antibodies to CD4 and CD25 (FITC-conjugated anti-mouse CD4 and APC-conjugated anti-mouse CD25, BD Biosciences). Cells were fixed and permeabilized prior to staining with Foxp3 (Tube 007), ROR-γt (Tube 008), T-bet (Tube 009), and GATA-3 (Tube 010) antibodies (PE-, BD Biosciences).

The splenocytes were stained with PerCP-Cy5.5-conjugated anti-mouse CD3e (PerCP-cy5.5-CD3e, eBioscience, San Diego, CA, USA), fluorescein isothiocyanate (FITC)-conjugated anti-mouse CD4 (FITC-CD4, BD Biosciences, San Jose, CA, USA), PE-cyanine7-conjugated anti-mouse CD8a (eBioscience), and APC-conjugated anti-mouse CD25 (Tube 011).

Unstained control (no antibody, Tube 001), FITC control (FITC-conjugated anti-mouse CD4 only, Tube 002), APC control (APC-conjugated anti-mouse CD25 only, Tube 003), PE control (PE-conjugated anti-mouse CD138 (BD Bioscience) only, Tube 004), and PerCP-Cy5.5 CD3 control (PerCP-Cy5.5-conjugated anti-mouse CD3e only, Tube 005) were also used for gating.

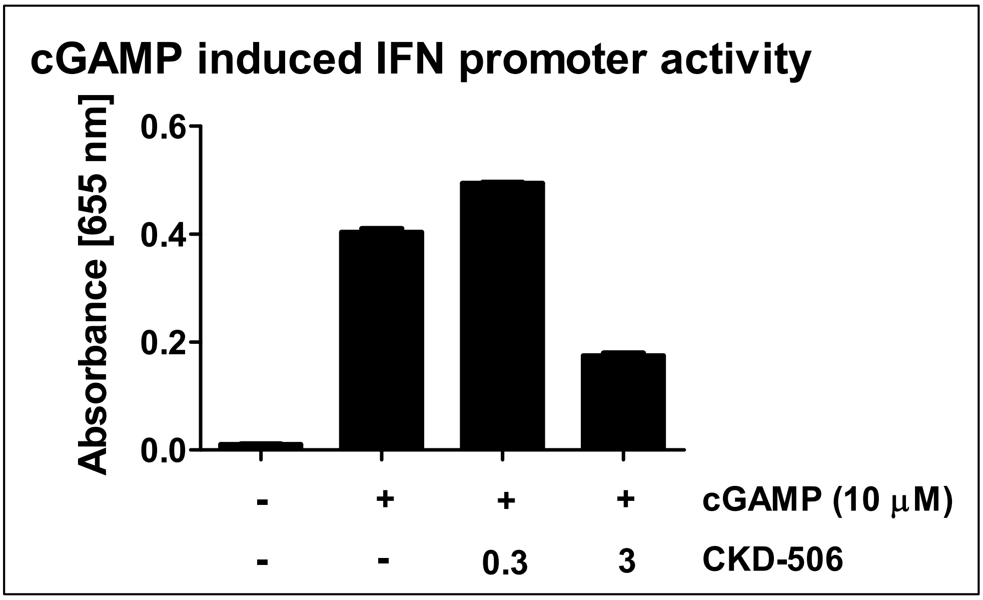

Supplementary Figure 3. To analyze the effect of CKD-506 on interferon (IFN) expression, a reporter assay was performed with the THP1-Blue ISG human monocyte cell line with an interferon regulatory factor (IRF)-inducible secreted alkaline phosphatase (SEAP) reporter construct (InvivoGen, USA). One hundred thousand THP1-Blue ISG cells in 200 μl were plated in a 96-well plate and stimulated in the presence of 10 μM cyclic guanosine monophosphate-adenosine monophosphate (cGAMP; STING agonist), a strong activator of IRF promoter, with the indicated concentration of CKD-506 for 24hr in a 37°C CO_2 incubator. The SEAP activity in the culture supernatant was measured with Flexstation (Molecular Devices, USA) after incubation with Quanti-Blue solution (InvivoGen, USA) for 15 min. Twenty microliters of culture supernatant was mixed with 180 ul of Quanti-Blue solution and incubated for 1hr at 37°C. CKD-506 at 3 μM significantly repressed IFN promoter activity induced by cGAMP, suggesting that CKD-506 represses the innate immune response of macrophages.
